# A Comparison of Three Protocols for Determining Barbell Bench Press Single Repetition Maximum, Barbell Kinetics, and Subsequent Measures of Muscular Performance in Resistance-Trained Adults

**DOI:** 10.3390/sports12120334

**Published:** 2024-12-03

**Authors:** Matthew T. Stratton, Austin T. Massengale, Riley A. Clark, Kaitlyn Evenson-McMurtry, Morgan Wormely

**Affiliations:** Basic and Applied Laboratory for Dietary Interventions in Exercise and Sport, Department of Health, Kinesiology, and Sport, University of South Alabama, Mobile, AL 36688, USA; atm1821@jagmail.southalabama.edu (A.T.M.); rc1922@jagmail.southalabama.edu (R.A.C.); kaa2222@jagmail.southalabama.edu (K.E.-M.); mcw2021@jagmail.southalabama.edu (M.W.)

**Keywords:** maximal strength assessment, resistance training, velocity-based training, rate of perceived exertion, average concentric velocity

## Abstract

Background: One repetition maximum (1RM) is a vital metric for exercise professionals, but various testing protocols exist, and their impacts on the resulting 1RM, barbell kinetics, and subsequent muscular performance testing are not well understood. This study aimed to compare two previously established protocols and a novel self-led method for determining bench press 1RM, 1RM barbell kinetics, and subsequent muscular performance measures. Methods: Twenty-four resistance-trained males (n = 12, 24 ± 6.1 years) and females (n = 12, 22.5 ± 5.5 years) completed three laboratory visits in a randomized crossover fashion. During each visit, a 1RM was established using one of the three protocols followed by a single set to volitional fatigue using 80% of their 1RM. A Sex:Protocol repeated measures ANOVA was used to determine the effects of sex and differences between protocols. Results: No significant differences were observed between the protocols for any measure, except for 1RM peak power (*p* = 0.036). Post hoc pairwise comparisons failed to identify any differences. Males showed significantly higher 1RM, average, and peak power (*p*s < 0.001), while females demonstrated a greater average concentric velocity (*p* = 0.031) at 1RM. Conclusions: These data suggest the protocol used to establish 1RM may have minimal impact on the final 1RM, 1RM barbell kinetics, and subsequent muscular endurance in a laboratory setting.

## 1. Introduction

The determination of maximal strength, defined as the maximal amount of force that an individual can exert on an external object with appropriate technique [1], is of vital importance to a wide range of individuals. For instance, concerning strength and conditioning practitioners, maximal strength is of great interest due to its relationship to improved athletic performance [1]. Additionally, measures of maximal strength may be used to aid in the prescription of training loads throughout a training cycle and to assess the effectiveness of previous training cycles [2,3]. Respective to clinicians, maximal strength is useful as a prognostic factor in those with cancer [4], aids in the screening of age-related changes in muscle function commonly associated with dynapenia [5], and has a strong relationship to all-cause mortality [6]. Exercise and sports science researchers also commonly include measures of maximal strength in investigations to assess the impact of various protocols, be it dietary [7], nutritional supplementation [8,9,10], or strength and conditioning [11], on changes in strength in cross-sectional or longitudinal settings. 

Maximal strength may be assessed in various ways, but the most straightforward method is to directly measure a single repetition maximum (1RM) for the given exercise. However, due to the challenges associated with 1RM testing—such as longer durations and the potential risk of injury—alternative methods for estimating 1RM have become commonplace. One such approach involves using previously validated prediction equations, which only require performing a single set to the point of volitional fatigue with a submaximal load [12,13,14,15]. However, prediction equations do not account for variation in repetition performance between athletes with the same relative percentage of 1RM [16,17,18] and have previously shown a decreased sensitivity to alterations in 1RM in response to training [15]. These issues with utilizing previously established prediction equations can potentially lead to either under or over-estimating 1RM [13,19,20]—negatively impacting exercise prescription–or misclassifying patients when estimated 1RM is used in diagnostic settings. 

Due to these limitations of the submaximal repetitions to fatigue method for estimating 1RM, others have sought to utilize load-velocity profiles. Previous findings have demonstrated a strong linear relationship (R^2^ ≥ 0.9) between 1RM and average concentric velocity (ACV) [21,22,23], with early investigations suggesting ACV at 1RM is relatively stable [22,24,25]. Conversely, more recent investigations have brought into question the validity of this method due to the large variation in ACV at an athlete’s 1RM [16]. A 2017 investigation by Banyard et al. demonstrated a coefficient of variation of 22.5% in ACV at a participant’s 1RM when performing the barbell back squat [16]. Additionally, a study by Garcia-Ramos et al. found that the previously established equations systematically underestimated 1RM when performing concentric-only bench press–the exercise utilized to develop the prediction equation used–and overestimated 1RM when a typical eccentric-concentric bench press was completed [21]. ACV has also been shown to vary at different percentages of 1RM between sexes [23,26] when performed during different times of day [27], training status [23,28], and age [29]. Lastly, an additional consideration when employing load velocity profiling for estimating 1RM is the need for a linear transducer to accurately assess bar velocity, which can be costly and requires trained personnel to operate the device and interpret the data. Due to all of the previously mentioned limitations, directly testing an athlete’s 1RM remains the ideal method in settings in which testing accuracy is paramount. 

The 1RM bench press test is widely recognized as the gold standard for measuring upper body maximal strength, primarily due to its popularity in athletic and laboratory settings, as well as the familiarity athletes and active individuals have with the exercise [30,31]. This familiarity helps minimize technique-related issues that could confound assessments of performance changes resulting from specific interventions. While considerable focus has been given to different methods for estimating 1RM based on previously tested values, there has been little research examining how the method used to directly assess 1RM impacts the final result and 1RM barbell kinetics commonly used in velocity-based training methods, such as ACV and peak power (PP). Often, when conducting exercise tests, the practitioner must consider the setting and the number of individuals being assessed. In laboratory settings in which only one participant may be undergoing testing at a time, a standardized protocol with specific rest times and load increases is common practice. However, applying similar protocols in large group settings, such as collegiate weight rooms with limited staff, can be challenging. This has led to interest in alternative methods that require less direct supervision and structure while still producing comparable results. One emerging trend in strength and conditioning settings is the use of participant choice to help autoregulate or select daily exercise sessions, which some research suggests may improve exercise adherence and enjoyment [32,33,34]. However, to the author’s knowledge, the application of such techniques to maximal strength testing has not yet been explored.

An additional concern when selecting testing methods is that in both field and research settings, 1RM testing is rarely conducted in isolation. Often, it is completed as part of a testing battery that includes other assessments, such as muscular endurance tests like repetitions to failure at a specific percentage of the calculated 1RM [7,9,10,30,35,36,37,38]. Therefore, it is important to consider, not only how a selected protocol may impact the desired 1RM, but any tests that may follow said testing as well. As such, the purpose of this investigation was to compare and contrast three different methods of assessing bench press 1RM—two previously established protocols (the standard National Strength and Conditioning Association (NSCA) protocol [39] and one developed by Klemp and colleagues [11]) along with a novel participant-led protocol on measures of bench press 1RM, ACV, PP, session RPE, and subsequent measures of muscular endurance. We hypothesize there will be no significant differences between the two previously established protocols, but the participant-led protocol will result in a significantly lower 1RM and session RPE.

## 2. Materials and Methods

### 2.1. Experimental Overview

**Figure 1:**
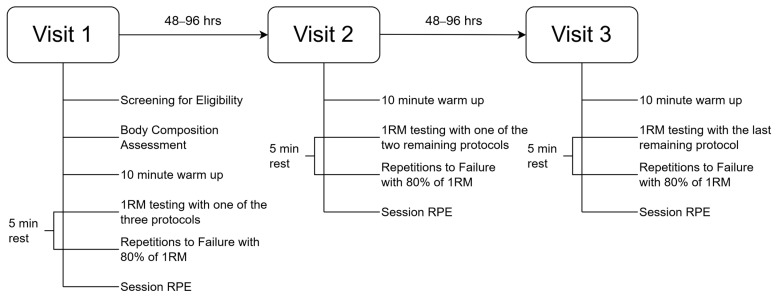
Timeline and overview of procedures.

**Figure 2:**
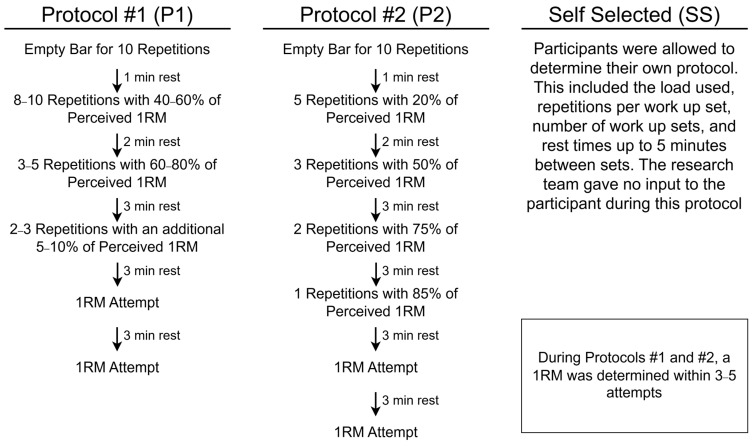
Description of the three methods utilized to determine barbell bench press 1RM. Protocol #1 (P1) was the guidelines set forth by the National Strength and Conditioning Association (NSCA) [39] while Protocol #2 (P2) was developed by Klemp and colleagues [11].

This counterbalanced, randomized, crossover investigation involved three laboratory visits. During each visit, participants completed a 10 min warm-up consisting of a 5 min general warm-up at a self-selected pace and intensity on a cycle ergometer and a 5 min self-led warm-up in which participants were allowed to complete exercises they would normally use prior to performing a barbell bench press. Following the completion of the warm-up, participants underwent one of three protocols to determine barbell bench press 1RM. Five minutes following the establishment of the 1RM visit, participants completed a single set to volitional fatigue utilizing 80% of the previously determined 1RM. Throughout all bench press sets, a linear transducer was connected to the barbell in order to record barbell kinetic data, including ACV, peak concentric velocity (PCV), average power (AP), and PP. A similar structure was repeated on visits two and three. One of the two remaining protocols was completed on visit two, and the final protocol was completed during visit three. The three protocols were completed in a randomized fashion. All visits were separated by 48–96 h and commenced at the same time of day ±1 h. An outline of the study timeline and procedures can be found in Figure 1 and Figure 2.

### 2.2. Participants

From September 2023 to March 2024, resistance-trained males and females were recruited to take part in this investigation. To be considered eligible to participate in this study, participants were required to resistance train at least twice per week for the 12 weeks prior to enrollment, with at least one of those sessions being an upper-body resistance training session, including either the flat barbell bench press or a variation (e.g., incline barbell bench press). Furthermore, participants were required to bench press ≥0.85× and ≥0.4× their body mass for males and females, respectively, during the initial visit. Conversely, potential participants were considered ineligible if they were currently using any anabolic substances (i.e., anabolic androgenic steroids, selective androgen receptor modulators, or prohormones); were currently injured in a way that would impact their ability to perform a flat barbell bench press, or reported any disease or medical condition in which performing high-intensity resistance training would be contraindicated. During the initial visit, all potential participants provided verbal and written consent after being informed of the study procedures and potential risks. 

#### Randomization and Determination of Sample Size

Since there were no similar investigations to power this study, the sample size was convenience-based and justified based on feasibility expectations [40]. Additionally, the sample size was chosen to aid randomization and ensure all potential orders were equally represented. The three protocols were randomly assigned so that each participant was able to complete each protocol in a randomized crossover fashion. Due to the inclusion of three protocols, there were six possible sequences in which participants could complete the investigation (i.e., P1-P2-SS, P1-SS-P2, P2-P1-SS, P2-SS-P1, SS-P1-P2, and SS-P2-P1). With the current sample size (24) and subgroups (12 males and 12 females), each sequence was represented four times in total and twice within each sub-group. The order in which the sequences were assigned to participants was determined using a randomized permuted block design function within the random R package [41].

### 2.3. Procedures

#### 2.3.1. Anthropometrics

During the initial visit—following the provision of informed consent and the conformation of compliance with all pretesting guidelines—the participant’s height (HM200P, Charder Medical, Taichung City, Taiwan), weight, and body fat percentage (Inbody H2On, InBody, Seoul, Republic of Korea) were assessed for descriptive purposes. This body composition device was chosen as it has previously shown acceptable concurrent validity with a four-compartment laboratory model [42]. Additionally, weight was collected to determine the minimum absolute bench press 1RM needed to be considered eligible for this study.

#### 2.3.2. Strength Testing

Prior to each visit, participants were required to abstain from any upper body resistance training for ≥48 h, strenuous exercise (anything greater than a brisk walk) for ≥24 h, and caffeine for ≥12 h. Following the verbal confirmation of compliance with these pretesting guidelines, participants began a 10 min period consisting of a generalized warm-up for 5 min at a self-selected pace and intensity on a cycle ergometer followed by 5 min of self-selected stretches or other forms of dynamic stretching or exercises before beginning the bench press. The stretches, sets, and repetitions for the self-selected exercises, along with intensity and rpm for the cycle ergometer, were recorded by the researchers and were then standardized as the participant’s warm-up for the following two visits to prevent any potential impact of differences in warm up on the subsequent 1RM testing. The self-selected warm-up period was included to allow the participants to more closely mimic what they would do in their own training, attempting to aid in the ecological validity of the investigation. 

Upon completion of the warm-up, a barbell bench press 1RM was determined using one of the following three methods. Protocol 1 (P1) was based on guidelines provided by the National Strength and Conditioning Association (NSCA) as described previously [36,39]. During this protocol, increases in weight between the prescribed percentages were selected by monitoring the RPE and ACV of the previous set, along with the experience of the researcher conducting the assessment. But all load increases fell within the prescribed percentage ranges set forth by the NSCA. Protocol 2 (P2) was drawn from the method previously described by Klemp and colleagues [11]. Finally, protocol three (SS) was a self-selected protocol that allowed participants to choose their warm-up intensities and repetitions leading up to their 1RM attempts without input from the research team. During P1 and P2, the resultant 1RM was determined within 5 attempts. Five minutes following the determination of the participant’s 1RM, a single set to volitional concentric failure was completed utilizing 80% of the previously established 1RM. A visual overview of the selected protocols can be found in Figure 2. 

The criterion for bench press quality was similar to previously described methods by Stratton and colleagues [7]. In short, the bench press was performed with the participant’s preferred grip width—this grip width was documented and repeated during visits 2 and 3—and the requirement that the bar contacted the chest and returned to the starting position. A repetition was only considered ‘good’ (i.e., counted) if the participant completed the repetition with feet on the floor and hips and upper back remaining in contact with the bench. All performance testing was completed with trained spotters and the supervision of an NSCA Certified Strength and Conditioning Specialist (CSCS). During each attempt, strong verbal encouragement was given from the research team. Throughout P1 and P2 testing visits, participants were not told what weight was loaded on the bar for each attempt with the goal of minimizing any potential psychological components of performance. However, as participants were allowed to dictate their own weight increases during all SS visits, they then had direct knowledge of the weight loaded on the bar and were no longer blinded. This unblinding was unfortunately necessary in order to fully compare the implications of a participant-led protocol. Calibrated weight plates were used during all sets to ensure the accuracy of the loaded weight. Participants were also asked not to look at the loaded weights whenever possible, and weight plates of the same diameter were used in order to minimize participants deducing the weight range by the size of the loaded plate. 

#### 2.3.3. Barbell Kinematics

In order to assess repetition quality, ACV, PCV, AP, and PP were monitored during all 1RM attempts via a linear transducer (Tendo Weightlifting Analyzer, TENDO Sports Machines, London, UK) and RTF. This transducer was positioned directly under the barbell and connected via an extended cable in a manner that created a 90-degree angle from the ground when the barbell was at the top of the repetition and the arms were fully extended. Data from every repetition was collected via TENDO Unit software, version 6.06 [43].

#### 2.3.4. Session RPE and Dietary Control

At the end of each visit, participants were asked to provide a rate of perceived exertion (RPE) for the entire session on a scale of 1–10, with 1 representing a minimally demanding workout and 10 corresponding to the hardest workout they could possibly complete. Lastly, at the end of the initial visit, participants completed a 24 h dietary recall with the aid of an International Society of Sports Nutrition Certified Sports Nutritionist (CISSN). The participants were then provided a copy of their recall and asked to replicate their intake for the 24 h before visits 2 and 3. Adherence to these guidelines was assessed at the start of visits 2 and 3. 

### 2.4. Statistical Analysis

A Sex (Male vs. Female) × Protocol (P1 vs. P2 vs. SS) repeated measures analysis of variance (ANOVA) utilizing the afex package [44] within R (v 4.2.1) [45] was used to assess any potential interactions and effects of sex and protocol. The normality of model residuals was examined via Shapiro–Wilk tests along with visual inspection of the quantile-quantile plot. However, in instances of normality violations in the residual values, the raw data were still utilized for analysis due to a number of reasons: (1) the frequent lack of normality improvement and/or differences in results upon implementation of data transformations; (2) the robust nature of the ANOVA against said violations (4) and (3) to aid in interpretability of the final results. In the event of an interaction, post hoc pairwise comparisons were completed with a Tukey adjustment. Simple main effects were assessed if no interactions were present. Partial eta squared ($\eta_{p}^{2}$) effect sizes were determined for all interactions and simple main effects. In the instance of a simple main effect of sex, a post hoc paired t-test was completed with a Tukey adjustment. Similarly, in the event of a simple main effect of protocol, a one-way repeated measures ANOVA was utilized with post hoc pairwise comparisons utilizing a Tukey adjustment. In the event of a violation of sphericity, a Greenhouse–Geisser correction was applied. Significance was accepted at *p* ≤ 0.05. 

## 3. Results

### 3.1. Participants

Twenty-four resistance-trained (6.0 ± 3.9 years) males (n = 12; 24.1 ± 6.1 years; 90.9 ± 10.6 kg; 180.6 ± 5.6 cm) and females (n = 12; 22.5 ± 5.5 years; 74.7 ± 15.2 kg; 163.3 ± 6.4 cm) agreed to take part in this investigation. Full participant characteristics can be found in Table 1. 

### 3.2. 1RM, Repetitions to Failure, and Session RPE

No Sex × Protocol interactions were seen for 1RM (*p* = 0.538, $\eta_{p}^{2}\;$= 0.024), RTF (*p* = 0.620, $\eta_{p}^{2}$ = 0.021), or session RPE (*p* = 0.951, $\eta_{p}^{2}\;$= 0.002). Additionally, no simple main effects of the protocol were detected for any of the aforementioned variables (*p*s > 0.302). However, a main effect of sex was noted for 1RM (*p* < 0.001, $\eta_{p}^{2}\;$= 0.756), with males displaying a significantly greater 1RM when collapsed across protocols (males: 111.4 ± 20.8 kg; females: 54.2 ± 10.9 kg; *p* < 0.001). Full results can be found in Table 2 and Table 3.

### 3.3. 1RM Barbell Kinetics (ACV, PCV, AP, PP)

Similarly to the previous data, no significant Sex × Protocol interactions were found. Conversely, the main effects of sex were detected for ACV (*p* = 0.031, $\eta_{p}^{2}$ = 0.194), AP (*p* < 0.001, $\eta_{p}^{2}$ = 0.498), and PP (*p* < 0.001, $\eta_{p}^{2}$ = 0.605). Post hoc paired samples t-tests with a Tukey adjustment revealed females displayed higher 1RM ACVs (males: 0.15 ± 0.06 m/s; females: 0.20 ± 0.06 m/s; *p* = 0.031). However, males recorded higher AP (males: 160.4 ± 54.8 W; females: 100.5 ± 32.2 W; *p* < 0.001) and PP (males: 454.6 ± 176.5 W; females: 208.9 ± 82.8 W; *p* < 0.001). Additionally, a main effect of the protocol was found for PP (*p* = 0.028, $\eta_{p}^{2}$ = 0.150). Post hoc pairwise comparisons with a Tukey adjustment failed to reveal any significant differences between protocols (*p* > 0.101). Interestingly, a significant difference was detected between protocols P1 and P2 (*p* = 0.034), and a trend was seen when P1 and P3 (*p* = 0.052) were compared. However, these values were no longer significant following the Tukey adjustment with *p* = 0.101 and *p* = 0.157, respectively. Initial RMANOVA results for all variables can be found in Table 2. 

### 3.4. RTF Barbell Kinetics (Max ACV, Min ACV, Velocity Drop off, Mean ACV)

A Sex × Protocol interaction was noted for minimum ACV (*p* = 0.034, $\eta_{p}^{2}$ = 0.156) and velocity drop-off (*p* = 0.003, $\eta_{p}^{2}$ = 0.235) throughout the single RTF set. Post hoc pairwise comparisons prior to corrections revealed that males displayed lower minimum ACVs throughout the set in P2 (0.16 ± 0.03 m/s) than in P1 (0.20 ± 0.06 m/s; *p* = 0.033), and females showed similar variations between P2 (0.19 ± 0.05 m/s) and SS (0.17 ± 0.04; *p* = 0.035) m/s protocols. However, neither of these comparisons were significant following the application of a Tukey correction with *p* = 0.248 and *p* = 0.260, respectively. With regard to velocity drop-off, similar applications of post hoc pairwise comparisons with a Tukey adjustment noted significant differences between males (61 ± 5.37%) and females (44.5 ± 14.2%; *p* = 0.012) following P2 along with females having less of a velocity drop off following P2 (44.5 ± 14.2%) when compared to SS (55.5 ± 8.0%; *p* = 0.049). Lastly, a main effect of sex was seen for mean ACV (*p* = 0.037, $\eta_{p}^{2}$ = 0.183), with males displaying greater mean ACV throughout the single RTF set than females (0.31 ± 0.05 m/s vs. 0.28 ± 0.04 m/s; *p* = 0.037) when collapsed across protocols following a Tukey correction. Full pairwise comparison results can be seen in Supplemental Tables S1 and S2. Full results are depicted in Table 4, Table 5 and Table 6. 

## 4. Discussion

The main findings of this study were that, in a laboratory setting, the protocol used to assess barbell bench press 1RM did not affect the resulting 1RM, 1RM barbell kinetics, session RPE, or subsequent repetitions to failure. Notably, this included instances where participants followed their own protocols without input from the research team (Table 2 and Table 3). Additionally, males demonstrated higher absolute 1RM, PP, and AP compared to females while females exhibited greater ACV than males at their respective 1RM. Interestingly, the current data refuted our hypothesis that the standardized protocols would result in similar findings (P1 vs. P2), but when participants were allowed to lead the session themselves (SS), it would result in decreased performance as the performance was similar among all protocols, not just P1 and P2. It must be noted that a significant main effect of protocol was seen for PP with pairwise comparisons revealing that P1 resulted in a reduced PP compared to both other protocols (P1: 291.3 ± 159 W; P2: 368.3 ± 211.5 W; SS: 350.8 W). However, these findings were no longer significant once a Tukey correction was applied. Additionally, females displayed a greater velocity drop-off following the SS protocol when compared to P2 (P2: 44.5 ± 14.2 m/s; SS: 55.5 ± 8.0 m/s; *p* = 0.049), though this finding did not extend to males. Lastly, males also showed a greater velocity drop-off than females throughout the single RTF set following P2. 

In a 2019 study, Ormsbee and colleagues reported that barbell bench press 1RM ACV was 0.15 m/s among college-aged “experienced bench pressers” (average experience of 4.7 ± 2.0 years) [28]. This is consistent with the 1RM ACV of 0.15 m/s found in the current study, suggesting that 0.15 m/s could be a useful benchmark when using estimated 1RM velocities to guide 1RM attempt selection in trained individuals when true 1RM ACV is unknown. Our additional finding that ACV at 1RM was higher in females (0.20 ± 0.06 m/s) than males (0.15 ± 0.06 m/s) similarly aligns with a previous investigation by Torrejon et al., who found that females performed 1RMs faster (0.19 ± 0.05 m/s) than their male counterparts (0.17 ± 0.04 m/s) when completing a smith machine bench press [23]. However, this is in contrast to a 2023 meta-analysis that indicated males may display higher ACVs than females at 30% and 70% of 1RM, though these differences disappeared at 90% of 1RM [46]. Balsalobre-Fernandez also similarly noted that males had higher ACVs across all percentages when performing the barbell military press, except at 100% of their 1RM [26]. These discrepant findings suggest that sex differences in load–velocity relationships may depend on the relative load used and exercise selected, although further research is needed to confirm or refute this hypothesis and explore any potential underlying mechanisms.

Similar to the previous work by Stratton et al. [18], sex differences were not present when performing the barbell bench press to failure at 80% of 1RM. This aligns with the findings of Maughan et al., in which no sex differences were apparent when performing forearm flexions with 80–90% of 1RM to momentary concentric failure [47]. Conversely, a 2022 investigation by Lewis and colleagues [48], noted females consistently were able to perform more dumbbell bicep curls with a 10RM than their male counterparts. These differences in findings likely stem from the relative intensity used. An individual’s 10RM has previously been estimated to equate to ~75% of their 1RM [39]. Maughan and colleagues [47] also noted similar sex differences to Lewis et al. [48] when performing exercises with < 80% of 1RM, thus suggesting that at higher relative intensities (i.e., ≥80% of 1RM), sex differences may no longer be apparent. An additional consideration is that the sex differences noted by Lewis et al. [48] and Maughan et al. [47], were found while using single joint exercises (single arm bicep curl and forearm flexion) while the present investigation used a large multi-joint compound exercise, suggesting the previously established sex differences may be ameliorated in large compound lifts. However, the present interesting finding that while total repetitions completed between sexes were not different, differences in average ACV and velocity drop-off suggest that some sex differences may still be apparent when also assessing repetition quality (i.e., velocity, etc.) at these higher percentages that may be missed if only examining surface measures of repetition total. Further investigations into how these differences may impact training outcomes and the use of VBT to guide training are warranted. 

The lack of differences in session RPE (P1: 7.0 ± 1.3; P2: 7.0 ± 1.6; SS: 6.6 ± 1.7; *p* = 0.302, $\eta_{p}^{2}$ = 0.023) is noteworthy due to previous studies that have shown individuals tend to report lower RPEs when exercising in their preferred manner, even when performing the same absolute work [27,49]. Reduced RPE, along with increased motivation during an exercise session, has been found to increase exercise enjoyment, leading to improved exercise adherence [33,34]. Furthermore, training methodologies in which participants can actively make choices to modulate the session to what they prefer at the moment, similar to the self-led protocol in the current investigation, have led to higher adherence in longitudinal settings [32]. However, in the present study, that was not the case. Upon completion of all three visits, of the twenty-four participants included in this investigation, only five (M: 1, F:4) reported they preferred the SS protocol, while the standardized protocols were essentially evenly preferred (P1: 9; P2: 10). 

An additional consideration when selecting a protocol for assessing any given metric is the time taken to complete the test. Notably, the three protocols required a comparable amount of time to complete (P1: 33.2 ± 5.5 min; P2: 37.0 ± 5.9 min; SS: 35.7 ± 6.3 min), with only slight variation in range ([min–max] P1: 17–44 min; P2: 28–53 min; SS: 20–48 min) as well. These data suggest that when participants are allowed to autoregulate their own maximal strength protocol, it may not impact or extend the time needed to perform the assessment to a significant degree. 

The present study is not without limitations. Firstly, this study was conducted solely using barbell bench press and within a laboratory environment. As such, these data may not extend out to other movements (e.g., barbell back squat, barbell deadlift, front squat, military press, etc.) and other field settings. Furthermore, due to the need to control the environment as much as possible, participants being tested in an unfamiliar setting and without the addition of self-selected music, etc., may have impacted the final result. Throughout the warmup sets for P1, percentage ranges were given as opposed to exact percentages. This potentially led to inconsistencies in relative increases in intensity while approaching any 1RM attempt and may have impacted the final result. However, this was performed in an attempt to best follow the recommended protocol as outlined by the NSCA [39] and how it is commonly represented in field and research settings [18,30,36], potentially aiding the ecological validity of its inclusion in this investigation. While during P1 and P2, every attempt was made to keep the participant blinded as to the amount of weight loaded onto the bar, it is still possible that some of the well-trained participants were still able to deduce the load, potentially causing psychological factors to be present. However, if this happened for one protocol, it was likely present during all three protocols, still allowing for equal comparison of the final 1RM and barbell kinetics. This was also a free-living study; while attempts were made to control for participant presentation at the start of the visit (requested exercise abstention and dietary logs), without direct participant observation during the time between visits, we cannot confirm that they were in fact followed. The potential impact of learning effects cannot be discounted. While the randomized nature and equal representation of protocol orders were used to help mitigate these impacts, they likely still played a factor in participant performance. Additionally, as the researchers had prior knowledge of the 1RMs found in previous visits, more precise attempt selection on later testing sessions may have impacted the lack of differences seen in the current study. Due to the absence of similar studies throughout the literature, a convenience sample was used that ensured equal representation of each randomization order. The lack of significant findings may partly stem from insufficient sample size and statistical power to detect relevant effects (e.g., the lack of differences in PP following a Tukey correction on post hoc pairwise comparisons). Future investigations should utilize these data to determine the appropriate sample sizes in addition to utilizing different protocols, populations (e.g., age groups, training statuses, etc.), individual exercises, and longer or varied testing batteries. Furthermore, additional factors may impact exercise test selection, such as muscle fiber composition, exercise technique, and participant anthropometrics. 

Due to the lack of significant differences between methods, practitioners should feel secure in utilizing protocols to determine bench press 1RM that fits with their clientele and situation. For example, when assessing large groups of athletes in which constant supervision to ensure a strict protocol is used may prove difficult, the athletes may be given agency to utilize their own methods. Conversely, in research settings in which every minute detail may be imperative to detect relevant differences, a standardized protocol may be developed that works with the facilities, time, and staff available. Lastly, our data suggest that if using ACV to aid in attempt selection for determining 1RM, universal 1RM targets may prove to be suboptimal, and considerations need to be made for aspects such as the sex of the trainee. 

## 5. Conclusions

The present investigation is the first to investigate the impact of the protocol used to assess bench press 1RM, 1RM barbell kinetics, and subsequent muscular endurance testing representative of protocols utilized throughout the literature. These data suggest that the impact of the protocol is likely minimal when assessing performance at maximal intensities. Additionally, the use of a standardized protocol did not result in a significant difference than if the participants were able to follow their own self-led protocol. 

## Figures and Tables

**Table 1:** Participant Characteristics.

Sample	Age (Years)	Height (cm)	Weight (kg)	BF% (%)	Years Trained (Years)
Males (n = 12)	24.1 ± 6.1	180.6 ± 5.6	90.9 ± 10.6	20.3 ± 5.5	7.8 ± 4.2
Females (n = 12)	22.5 ± 5.5	163.3 ± 6.4	74.7 ± 15.2	31.4 ± 9.4	4.1 ± 2.6
Full Sample (n = 24)	23.3 ± 5.7	172.0 ± 10.6	82.8 ± 15.3	25.9 ± 9.5	6.0 ± 3.9

All data are presented as means ± standard deviation. 1RM: one repetition maximum; years: years; cm: centimeters; kg: kilograms; BF%: body fat percentage.

**Table 2:** Performance results.

	1RM (kgs)			RTF (Repetitions)			RPE (A.U.)		
Protocol	P1	P2	SS	P1	P2	SS	P1	P2	SS
Males (n = 12)	111.5 ± 21.6	112.0 ± 21.7	110.6 ± 21.0	6.7 ± 1.4	7.1 ± 1.6	6.7 ± 1.4	7.4 ± 0.9	7.3 ± 1.5	7.0 ± 1.2
Females (n = 12)	54.2 ± 10.4	54.2 ± 11.6	54.3 ± 11.6	5.9 ± 1.6	5.8 ± 2.0	5.9 ± 1.6	6.6 ± 1.6	6.6 ± 1.6	6.1 ± 2.0
Full Sample (n = 24)	82.8 ± 33.6	83.1 ± 34.1	82.5 ± 33.2	6.3 ± 1.5	6.5 ± 1.9	6.3 ± 1.5	7.0 ± 1.3	7.0 ± 1.6	6.6 ± 1.7

All data are presented as means ± standard deviation. 1RM: one repetition maximum; RTF: repetitions to failure at 80% of 1RM; RPE: rate of perceived exertion utilizing a 1–10 scale with a higher score corresponding to an increased perceived difficulty of the workout; kgs: kilograms; A.U.: arbitrary units.

**Table 3:** Initial RMANOVA results for all 1RM variables.

	1RM		RTF		RPE		ACV		PCV		AP		PP	
Comparison	*p*	$\eta_{p}^{2}$	*p*	$\eta_{p}^{2}$	*p*	$\eta_{p}^{2}$	*p*	$\eta_{p}^{2}$	*p*	$\eta_{p}^{2}$	*p*	$\eta_{p}^{2}$	*p*	$\eta_{p}^{2}$
Sex	<0.001 *	0.756	0.130	0.101	0.128	0.102	0.031 *	0.194	0.661	0.009	<0.001 *	0.498	<0.001 *	0.605
Protocol	0.638	0.017	0.500	0.031	0.302	0.023	0.529	0.027	0.099	0.107	0.660	0.018	0.036 *	0.150
Sex × Protocol	0.538	0.024	0.620	0.021	0.951	0.002	0.739	0.012	0.372	0.042	0.806	0.009	0.145	0.087

RMANOVA: repeated measures analysis of variance; 1RM: one repetition maximum; RTF: repetitions to failure at 80% of 1RM; RPE: rate of perceived exertion; ACV: average concentric velocity; PCV: peak concentric velocity; AP: average power; PP: peak power; *: significant difference defined as *p* < 0.05.

**Table 4:** Barbell kinetic 1RM results.

	ACV (m/s)			PCV (m/s)			AP (W)			PP (W)		
Protocol	P1	P2	SS	P1	P2	SS	P1	P2	SS	P1	P2	SS
Males (n = 12)	0.15 ±0.05	0.17 ±0.07	0.15 ±0.06	0.34 ±0.10	0.43 ±0.13	0.39 ±0.13	154.1 ±52.8	169.8 ±43.4	157.3 ±68.8	392.2 ±147.1	527.9 ±175.5	473.8 ±191
Females (n = 12)	0.18 ±0.07	0.20 ±0.07	0.20 ±0.05	0.35 ±0.12	0.37 ±0.09	0.39 ±0.07	96.2 ±39.6	101.7 ±34.2	103.8 ±23	190.3 ±94.7	208.8 ±84.9	227.8 ±69.8
Full Sample (n = 24)	0.17 ±0.06	0.18 ±0.07	0.17 ±0.06	0.35 ±0.11	0.40 ±0.11	0.39 ±0.10	125.1 ±54.4	135.8 ±51.7	130.5 ±57.1	291.3 ±159	368.3 ±211.5	350.8 ±188.6

All data are presented as means ± standard deviation. ACV: average concentric velocity; PCV: peak concentric velocity; AP: average power; PP: peak power; W: watts; m/s: meters per second.

**Table 5:** Barbell kinetic repetitions to failure at 80% of 1RM results.

	Max ACV (m/s)			Min ACV (m/s)			Velocity Drop Off (%)			Mean ACV (m/s)		
Protocol	P1	P2	SS	P1	P2	SS	P1	P2	SS	P1	P2	SS
Males (n = 12)	0.40 ±0.06	0.42 ±0.06	0.41 ±0.06	0.20 ±0.06	0.16 ±0.03	0.16 ±0.03	49.6 ±14.3	61.0 ±5.4	59.3 ±11.1	0.31 ±0.05	0.31 ±0.05	0.30 ±0.04
Females (n = 12)	0.39 ±0.05	0.35 ±0.07	0.37 ±0.08	0.17 ±0.04	0.19 ±0.05	0.17 ±0.04	56.7 ±12.3	44.5 ±14.2	55.5 ±8.0	0.28 ±0.03	0.27 ±0.05	0.27 ±0.05
Full Sample (n = 24)	0.39 ±0.06	0.39 ±0.07	0.39 ±0.07	0.18 ±0.05	0.18 ±0.04	0.16 ±0.04	53.2 ±13.5	52.8 ±13.4	57.4 ±9.7	0.30 ±0.04	0.29 ±0.05	0.29 ±0.05

All data are presented as means ± standard deviation. ACV: average concentric velocity; m/s: meters per second; velocity drop off was calculated as (Max ACV–Min ACV)/Max ACV × 100.

**Table 6:** Initial RMANOVA results for all RTF barbell kinetic variables.

	Max ACV		Min ACV		Velocity Drop Off		Mean ACV	
Comparison	*p*	$\eta_{p}^{2}$	*p*	$\eta_{p}^{2}$	*p*	$\eta_{p}^{2}$	*p*	$\eta_{p}^{2}$
Sex	0.108	0.113	0.944	0.000	0.128	0.102	0.037 *	0.183
Protocol	0.845	0.008	0.235	0.064	0.288	0.055	0.444	0.036
Sex × Protocol	0.058	0.122	0.034 *	0.156	0.003 *	0.235	0.799	0.010

RMANOVA: repeated measures analysis of variance; repetitions to failure at 80% of 1RM; *: significant difference defined as *p* < 0.05.

## Data Availability

Individual data are available upon request to the corresponding author.

## Supplementary Materials

The following supporting information can be downloaded at: https://www.mdpi.com/article/10.3390/sports12120334/s1, Table S1: Post hoc comparisons for a Sex × Protocol interaction for min ACV; Table S2: Post hoc comparisons for a Sex × Protocol interaction for velocity drop-off.
