# Supplementary material for: A Comparison of Three Protocols for Determining Barbell Bench Press Single Repetition Maximum, Barbell Kinetics, and Subsequent Measures of Muscular Performance in Resistance-Trained Adults
Source: Sports (Basel). 2024 Dec 3;12(12):334. doi: 10.3390/sports12120334 (PMC11679921; doi:10.3390/sports12120334)
Supplement: Supplementary file 1 [file sports-12-00334-s001.zip › sports-3325017-supplementary.pdf]

Table S1 Post hoc comparisons for a Sex × Protocol interaction for min ACV

Comparison									
**Protocol**	**Sex**	**Protocol**	**Sex**	**Mean Difference**	**SE**	**df**	**t**	**p**	**P_{tukey}**
P1	Males	- P1	Females	0.03417	0.0199	22.0	1.7183	0.100	0.535
		- P2	Males	0.03583	0.0158	22.0	2.2700	0.033	0.248
		- P2	Females	0.00833	0.0179	22.0	0.4668	0.645	0.997
		- SS	Males	0.03750	0.0184	22.0	2.0342	0.054	0.356
		- SS	Females	0.03333	0.0179	22.0	1.8637	0.076	0.449
	Females	- P2	Males	0.00167	0.0179	22.0	0.0934	0.926	1.000
		- P2	Females	-0.02583	0.0158	22.0	-1.6365	0.116	0.585
		- SS	Males	0.00333	0.0179	22.0	0.1864	0.854	1.000
		- SS	Females	-0.00083	0.0184	22.0	-0.0452	0.964	1.000
P2	Males	- P2	Females	-0.02750	0.0156	22.0	-1.7674	0.091	0.505
		- SS	Males	0.00167	0.0112	22.0	0.1494	0.883	1.000
		- SS	Females	-0.00250	0.0156	22.0	-0.1603	0.874	1.000
	Females	- SS	Males	0.02917	0.0156	22.0	1.8701	0.075	0.445
		- SS	Females	0.02500	0.0112	22.0	2.2406	0.035	0.260
SS	Males	- SS	Females	-0.00417	0.0156	22.0	-0.2665	0.792	1.000

ACV: average concentric velocity; SE: standard error; df: degrees of freedom; p: uncorrected p-value; P_{tukey}: p-value following a Tukey correction.

Table S2 Post hoc comparisons for a Sex × Protocol interaction for velocity drop-off

Comparison									
**Protocol**	**Sex**	**Protocol**	**Sex**	**Mean Difference**	**SE**	**df**	**t**	**p**	**P_{tukey}**
P1	Males	- P1	Females	-7.13	5.45	22.0	-1.308	0.204	0.778
		- P2	Males	-11.40	4.82	22.0	-2.367	0.027	0.211
		- P2	Females	5.12	4.94	22.0	1.036	0.311	0.901
		- SS	Males	-9.67	5.14	22.0	-1.879	0.074	0.440
		- SS	Females	-5.93	4.76	22.0	-1.247	0.226	0.809
	Females	- P2	Males	-4.28	4.94	22.0	-0.866	0.396	0.951
		- P2	Females	12.24	4.82	22.0	2.542	0.019	0.155
		- SS	Males	-2.54	4.76	22.0	-0.534	0.599	0.994
		- SS	Females	1.19	5.14	22.0	0.232	0.819	1.000
P2	Males	- P2	Females	16.52	4.37	22.0	3.779	0.001	**0.012***
		- SS	Males	1.73	3.54	22.0	0.490	0.629	0.996
		- SS	Females	5.47	4.17	22.0	1.312	0.203	0.776
	Females	- SS	Males	-14.78	4.17	22.0	-3.548	0.002	**0.020***
		- SS	Females	-11.05	3.54	22.0	-3.121	0.005	**0.049***
SS	Males	- SS	Females	3.73	3.95	22.0	0.945	0.355	0.930

SE: standard error; df: degrees of freedom; p: uncorrected p-value; P_{tukey}: p value following a Tukey correction; *: significant difference defined as $p \leq 0.05$.
